# Polycyclic Aromatic Hydrocarbons (PAHs) in Grilled Marshmallows

**DOI:** 10.3390/molecules29133119

**Published:** 2024-06-30

**Authors:** Maciej Maciejczyk, Beata Janoszka, Magdalena Szumska, Beata Pastuszka, Sławomir Waligóra, Aleksandra Damasiewicz-Bodzek, Agnieszka Nowak, Krystyna Tyrpień-Golder

**Affiliations:** 1 Department of Chemistry, Faculty of Medical Sciences in Zabrze, Medical University of Silesia, Jordana 19, 41-808 Katowice, Poland; bjanoszka@sum.edu.pl (B.J.); mszumska@sum.edu.pl (M.S.); swaligora@sum.edu.pl (S.W.); aleksandra.bodzek@sum.edu.pl (A.D.-B.); agnieszkanowak@sum.edu.pl (A.N.); 2 Research and Implementation Center Silesia LabMed, Medical University of Silesia, Medyków 18, 40-752 Katowice, Poland; betty.p2308@gmail.com

**Keywords:** carcinogenic PAHs, marshmallows, chromatography

## Abstract

The aim of this study was to assess potential health risks among children and adolescents consuming various grilled marshmallows using a survey and to determine polycyclic aromatic hydrocarbons (PAHs) in these food products. PAH analysis in grilled marshmallows included a dilution stage with deionized water and liquid–liquid extraction with cyclohexane and solid-phase extraction (SPE). PAH fractions were initially analyzed via high-performance thin-layer chromatography, and PAH concentrations were determined via gas chromatography with a tandem mass detector using the selective reaction monitoring (SRM) mode. This study on the consumption of grilled marshmallows was conducted among approximately 300 children and adolescents. The preliminary results indicated that “raw” marshmallows did not contain PAHs. However, the obtained data suggested the exposure of young people to carcinogenic PAHs from grilled marshmallows (63.5% of them consumed marshmallows). Carcinogenic benzo(a)pyrene (BaP) was determined in all samples. The profile of PAH concentrations in the extracts isolated from various grilled types of marshmallows was similar (r^2^ > 0.8000), regardless of the grilling method. Compared to the white sugar confection, higher concentrations of PAHs were determined in multicolored marshmallows. The lack of social awareness about exposure to carcinogenic substances is alarming.

## 1. Introduction

Marshmallows are one of the earliest confections known to humankind, and they consist of several ingredients that can be divided into two main groups, i.e., sweeteners and emulsifiers [1]. Corn syrup, as a sweetener, is used more commonly than sugar because it increases solubility and prevents crystallization. Corn starch, modified food starch, water, gum, and gelatin with solubilized substances, sunflower oil and β-carotene are structure-forming agents, and whipped egg whites can be used in various proportions [1,2]. Emulsifying agents maintain fat distribution and provide the aeration that makes marshmallows puffy [3,4]. Gum obtained from plants can act as an emulsifier in marshmallows or a gelling agent [5]. Natural substances, such as roselle (*Hibiscus sabdariffa*) and black chokeberry, are used as colorants [2], and artificial dyes are often added. Additionally, most marshmallows contain natural and/or artificial flavors [5].

Although marshmallows do not have any special nutritional value, they are eagerly consumed by children and teenagers, particularly at parties. They are grilled over the bonfire, in the oven, or over a lit candle.

Currently, grilling sophisticated dishes is becoming a way of spending time for many people. In recent years, a unique trend has emerged in Poland where marshmallows are consumed in their original form. They can also be grilled for a delightful taste experience. The fashion for grilling marshmallows, especially over the bonfire, gained popularity in Poland due to foreign cartoons for children. Roasted marshmallows became a treat during outdoor gatherings, picnics and bonfire activities. Skewering marshmallows for roasting over an open flame has gained popularity as a social tradition, particularly among friends and family. However, as with any food consumption habit, it is important to examine the potential health implications associated with the consumption of grilled marshmallows.

While marshmallows are generally considered safe for consumption, the grilling process may introduce changes in their chemical composition, leading to the formation of potentially harmful compounds. It is suspected that grilled marshmallows may be a source of exposure to harmful compounds, including carcinogenic agents, such as polycyclic aromatic hydrocarbons (PAHs) and their derivatives.

Apart from natural sources, tobacco smoking and automotive and industrial emissions, the main sources of PAH contamination include food processing, such as curing, smoking, frying and grilling [6,7]. PAHs in food are formed when organic substances are exposed to high temperatures, such as during grilling (barbecuing).

Thermal processing leads to the formation of high-mass brown compounds within the advanced stages of the Maillard reaction [6]. Heating marshmallows over a fire causes sugar caramelization, a chemical reaction at high temperatures that produces a brown color and toasted flavor [7]. For marshmallows that are very slowly roasted, the reaction between sugars and amino acids in gelatin, which is known as the browning reaction, might be the reason for the golden color and general deliciousness, and this depends on the ratio of sucrose to corn syrup [8,9,10]. Raw foods usually do not contain high levels of PAHs. However, as with any food preparation method, potential health hazards need to be considered. There is insufficient knowledge about harmful substances in grilled marshmallows, as evidenced by a very limited number of papers on the subject. At the same time, young people have access to many recipes [11] and instructions on the Internet on how to grill marshmallows. Moreover, many supermarkets also sell ready-to-grill marshmallows.

Roasting marshmallows can lead to the formation of compounds that may have adverse effects on human health. For instance, when organic substances, such as sugars and proteins, present in marshmallows are subjected to intense heat, they can undergo chemical reactions that produce heterocyclic amines (HCAs) and polycyclic aromatic hydrocarbons (PAHs). However, the effect of the properties of the amino acids and carbohydrates on the production of PAHs is poorly understood [12].

Many epidemiological studies have demonstrated that exposure to PAHs is associated with increasing rates of some diseases, including cancer and cardiovascular, respiratory and gastrointestinal disorders [6,13,14]. In addition, they have mutagenic, teratogenic and endocrine-disrupting properties [15].

Food is one of the main sources of human exposure to PAHs [16,17]. However, in many countries, the main source of PAHs in children is indoor and outdoor air pollution [18]. According to Commission Regulation (EU) No 835/2011 of 19 August 2011 amending Regulation (EC) No 1881/2006 as regards maximum levels for PAHs in foodstuffs, the established maximum levels of benzo(a)pyrene (BaP) and PAH4 (i.e., the sum of BaP, benzo(a)anthracene, chrysene and benzo(b)fluoranthene) in products for infants and young children should be the lowest of all food products covered by the regulation. These products include the following: processed cereal-based foods and baby food for infants and young children (1), infant formulae and follow-on formulae, including infant milk and follow-on milk (2), as well as dietary foods for special medical purposes intended specifically for infants (3). The concentrations of BaP and PAH4 in these products should not exceed 1 μg/kg [19].

Many questionnaire studies are carried out on the nutrition of children and adolescents. However, we did not find questions about the consumption of grilled marshmallows. A cross-sectional survey of 132,489 children aged six to nine years, their parents and carers from 23 countries as part of the WHO European Childhood Obesity Surveillance Initiative (COSI) analyzed the responses to many questions. The research stressed the urgent need to create a healthier food environment and health system to promote healthy diets and support surveillance of childhood nutrition and obesity. However, it did not mention the forms of sweet consumption, such as grilled marshmallows [20].

One of the priority tasks for the future is to follow the principles of healthy eating for children in Europe in line with the WHO recommendations and to regulate the marketing of unhealthy foods [21]. Nevertheless, ready-to-grill marshmallows are available in many supermarkets. In addition, special attention should be paid to 13–17-year-olds because they made more unhealthy choices than younger children [22].

Since PAHs are hydrophobic, they can accumulate in the food chain. After ingestion, PAHs may undergo metabolic activation in human mammalian cells, and their products may be the result of their biological activity [13]. It has been confirmed that the following are biologically active PAHs that commonly occur in food products: acenaphthylene, acenaphthene, pyrene (Pyr), fluorene (Flu), naphthalene (Nap), phenanthrene (Phe), benzo(b)fluoranthene (BbFl), benzo(k)fluoranthene (BkFl), anthracene (Ant), fluoranthene (Flt), benzo(a)anthracene (BaA), chrysene (Chry), dibenzo(a,h)anthracene (DiBahA), indeno(1,2,3-c,d)pyrene (IP), benzo(ghi)perylene (BghiP) and benzo(a)pyrene (BaP) [23]. According to the United States Environmental Protection Agency (US-EPA), the above PAHs should be analyzed in food products [24,25].

The International Agency for Research on Cancer (IARC) has classified many of these compounds as probably carcinogenic (IARC group 2A), possibly carcinogenic (group 2B), or carcinogenic to humans (e.g., BaP) (group 1) [26]. The carcinogenic BaP is not a suitable marker for the presence of PAHs in food, according to the Scientific Panel on Contaminants in the Food Chain of the European Food Safety Authority (EFSA) [27]. Instead, the panel recommended using a system of four compounds (PAH4), including BaP, BaA, BbFl and Chry, or eight compounds (PAH8), i.e., PAH4, BkFl, BghiP, DiBahA and IP [19].

In their studies, Zelinkova and Wenz [25] and Bulanda and Janoszka [6] presented detailed information on the analysis methods of PAHs and their occurrence in food and regulatory and risk management aspects. The characteristics of various types of food according to the PAH content and the discussion about the impact of these compounds on the human body, taking into account methods for assessing human exposure to PAHs, were reported by Maciejczyk et al. [28].

PAH concentrations in food are usually low and are expressed in ng/g [6,28,29]. The determination of PAHs in food requires the use of multi-stage analytical procedures. However, the extraction of PAHs from food is crucial at the sample preparation stage [6,30].

Following the homogenization of a foodstuff, PAHs are extracted using different techniques prior to clean-up and purification [6,30]. Many analytical techniques have been performed to detect and measure PAHs in different food products. PAH concentrations are most often identified and quantified using gas chromatography equipped with mass spectrometry or a flame ionization detector (GC-MS/FID) and high-performance liquid chromatography equipped with ultraviolet or fluorescence detectors (HPLC-UV/FLD) or coupled to mass spectrometry (MS) or MS/MS [6,12,28]. Fluorescence detection is the most widely used technique for PAH detection after separation by liquid chromatography (LC).

Understanding the impact of grilled marshmallows on human health, especially on adolescents, requires a comprehensive analysis of their chemical composition.

To the best of our knowledge, there have been no studies on the content of PAHs in grilled marshmallows. Therefore, the aim of this study was to assess the potential health risks among children and adolescents consuming various grilled marshmallows using a survey and to determine PAHs in these food products.

## 2. Results and Discussion

Our study used an original questionnaire for surveys aimed at assessing how much and how often children aged 8 to 18 consumed grilled marshmallows. It also estimated the awareness of the harmful effects of these products among children. The questionnaire consisted of 17 single/multiple choice and open-answer questions. Completing the questionnaire on the Internet was voluntary and anonymous. In total, 329 children, 62% (*n* = 204) girls and 38% (*n* = 125) boys, participated in the survey. The mean (SD) age of the respondents was 13.8 (±2.7) years. A total of 58% (*n* = 191) of the participants lived in the city, while 42% (*n* = 138) in the countryside. A total of 95.1% (*n* = 313) of the respondents knew what marshmallows were, and 63.5% (*n* = 209) confirmed eating marshmallows. Furthermore, all of them ate grilled marshmallows. It is important to note that 78% (*n* = 163) of the respondents consumed them several times a year, while 22% (*n* = 46) consumed them once a month or more often.

As regards the grilling method, 84.2% (*n* = 176) of the respondents grilled marshmallows over the bonfire, 21.5% (*n* = 45) prepared marshmallows on the grill, 7.2% (*n* = 15) grilled marshmallows in the oven and over the gas burner and 4.3% (*n* = 9) in the fireplace. A total of 49.8% (*n* = 104) of the respondents grilled only white marshmallows, 10.1 (*n* = 21) grilled only colored marshmallows and 40.2% (*n* = 84) grilled both of them.

Although 71.7% (*n* = 195) of children ate grilled marshmallows, 28.8% (*n* = 92) knew and 45.9% (*n* = 147) believed that the consumption of grilled marshmallows was harmful to health. Only 17.8% (*n* = 57) of the responders did not know if eating them could have some adverse effect on their health. The alarming fact is that 76.5% (*n* = 218) of respondents’ parents allowed their children to eat grilled marshmallows. Therefore, in addition to improving nutritional habits (at school, home and in other places where children are), actions involving not only parents but also other adults caring for children are warranted [20].

In the process of roasting marshmallows, we noticed that their color changed from caramel through light brown to dark brown. This finding allowed us to suspect that the browning reaction had occurred, which could result in the formation of PAHs [10].

Therefore, the preliminary chromatographic analysis of PAH fractions isolated by SPE from cyclohexane extracts of grilled and non-grilled marshmallows was performed using the HPTLC technique.

**Figure 1:**
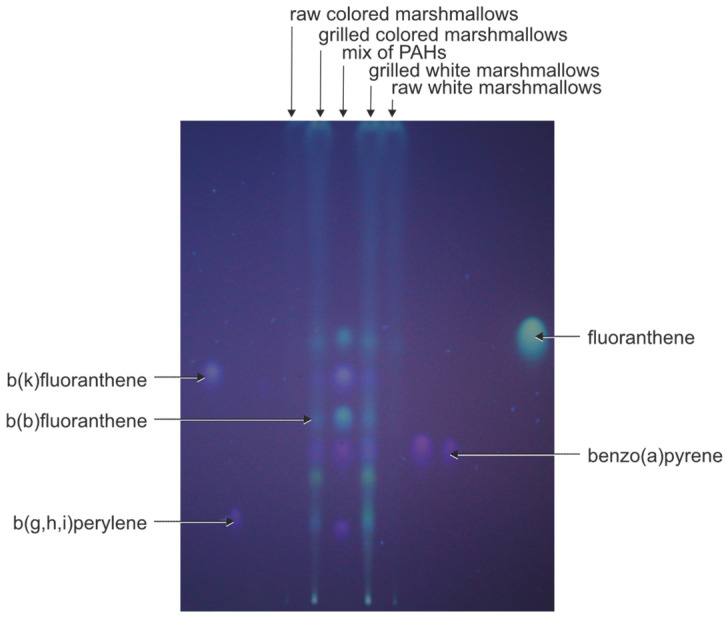
HPTLC results of separation of fraction isolated from raw and bonfire-grilled marshmallows (HPTLC Nano-SIL-PAH F_254_ plates, the nano-silica layer impregnated with the caffeine, developed with dichloromethane, and observed under UV light at λ = 366 nm).

The chromatographic plate was exposed to UV light. The comparison of the location and the color of spots of standard PAHs and PAH fractions isolated from grilled marshmallows extracts showed that PAHs were present in both white and colored grilled marshmallows. No PAHs were detected in raw marshmallow extracts. The results of the HPTLC analysis of grilled marshmallow extracts of PAHs are given in Figure 1. The analysis showed that BaP was present in the extracts isolated from grilled marshmallows.

Using densitometric detection, the same procedure was applied to determine the presence of PAHs in water samples [31]. The results obtained by HPTLC were confirmed by GC-MS/MS analysis.

For a more accurate qualitative and quantitative PAH analysis, many analytical techniques have been used in various food products, including GC-MS or gas chromatography equipped with mass spectrometry or flame ionization detector (GC-FID) and high-performance liquid chromatography equipped with ultraviolet or fluorescence detectors (HPLC-UV/FLD) [32]. GC-MS has become widely used in the analysis of PAHs in food due to the high selectivity of the MS detector. The frequency of use of tandem mass spectrometry (MS/MS) is increasing, providing more specific mass fragments, which increases the specificity and sensitivity of this technique [33].

The limits of detection (LOD) based on the signal-to-noise ratio (S/N = 3) were set for PAHs, which were considered possible to occur in grilled marshmallows. The limit of quantification (LOQ) was taken as three times the LOD (Table 1).

Excellent recovery values ranging from 60.70% (for IP and DiBahA, spiked with 20 ng/g of marshmallows) to 95.3% (for Chry, 40 ng/g of marshmallows) were obtained with satisfactory repeatability, on average, from 0.42% to 10.47% for PAHs in grilled marshmallows using a multi-step analytical scheme with the GC-MS/MS technique.

**Figure 2:**
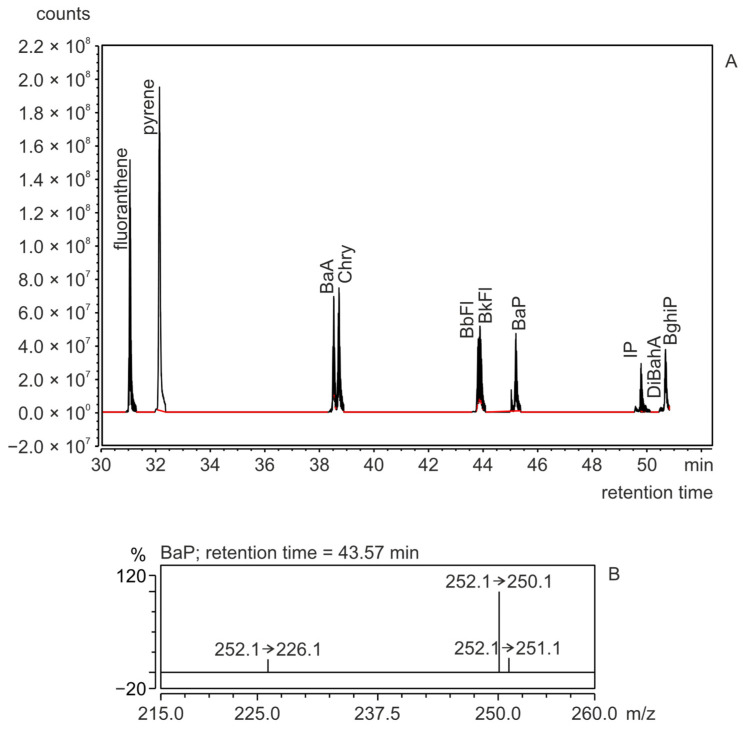
The GC-MS/MS chromatogram for the PAHs fraction isolated from colored marshmallows grilled over the bonfire (**A**) and an example SRM mass spectrum of BaP determined in this fraction (**B**).

The exemplary GC-MS/MS chromatogram of the PAH fraction isolated from colored marshmallows grilled over the bonfire and an example mass spectrum of determined BaP in this fraction (SRM) are given in Figure 2A,B. The concentrations of PAHs in grilled and non-grilled marshmallows [ng/g] are given in Table 2.

**Figure 3:**
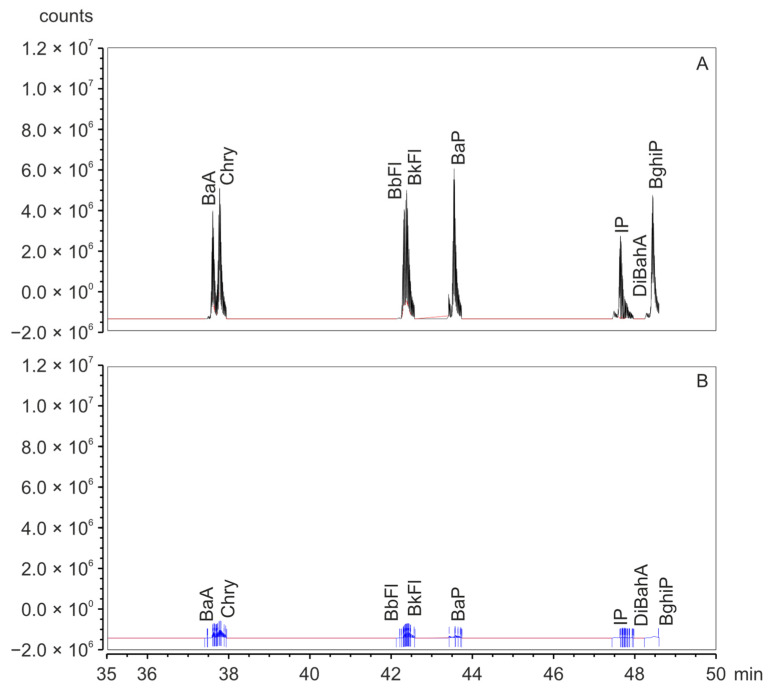
The fragment of GC-MS/MS chromatograms recorded for PAHs fraction isolated from grilled (colored, classic) (**A**) and non-grilled (raw, classic) marshmallows (**B**).

Studies have confirmed that the presence of sugars in food, especially glucose, may influence the type and increase the concentration of PAHs produced during the thermal decomposition of amino acids [34,35]. On the other hand, marshmallows are made not only from sugar but also from egg whites. Model tests have shown that PAHs can be formed from α-amino acids, fats, fatty acids, cholesterol and plant sterols. These compounds are easily fragmented at high temperatures (e.g., above 200 °C) during pyrolysis, and free radicals can form PAHs during pyrosynthesis [36,37]. Figure 3 shows the comparisons of chromatograms of PAH8 for grilled and non-grilled marshmallows.

Based on these data, it can be concluded that PAHs were not present in non-grilled marshmallows or were at the detection limit.

The estimation of European population exposure showed that 20% of the population was exposed to ambient BaP levels exceeding the norm given in the EU directive 2004/107/EC [38]. The presence of PAHs in other elements of the environment and in food increases the exposure to BaP and other carcinogenic PAHs [39]. According to the EFSA, carcinogenic BaP is not the only marker for the presence of PAHs in food [28]. However, Table 3 shows that the concentration of BaP in grilled marshmallows was the highest and, in many cases, exceeded the value for children (1 μg/kg). Table 3 shows that the BaP-PAH8 ratio in grilled marshmallows ranged from 1.26 to 5.84.

**Figure 4:**
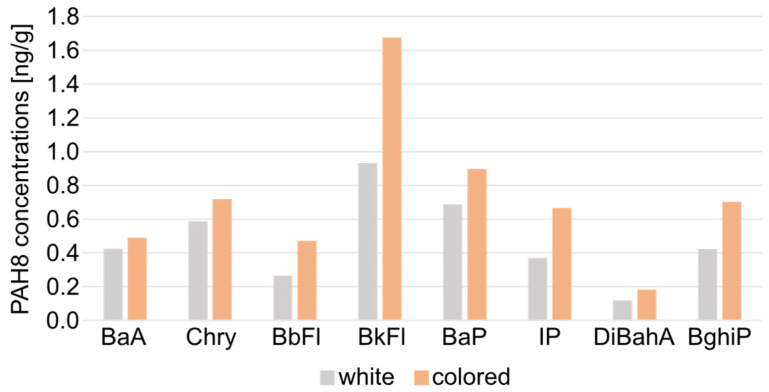
Comparison of PAH8 concentrations [ng/g] in white and colored grilled marshmallows.

In addition, the influence of marshmallow composition on the presence of PAH8 is given in Figure 4.

The PAH8 concentrations in bonfire-grilled colored marshmallows were higher in each case than in the extracts isolated from white marshmallows (on average 53.11 ± 26.90% more). One explanation for this phenomenon is the composition of colored marshmallows, especially the presence of dyes.

**Figure 5:**
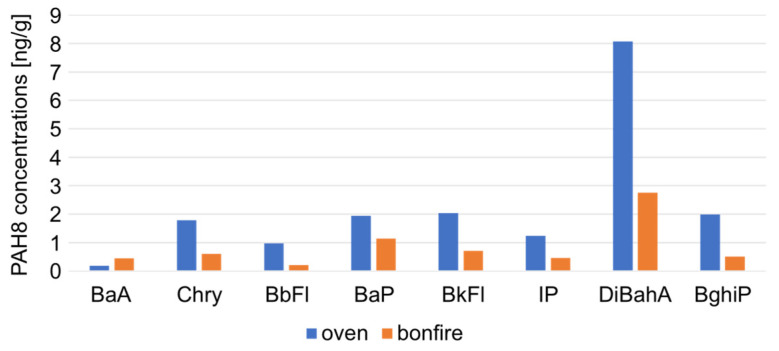
Concentrations of PAH8 in white marshmallows grilled in an electric oven and over the bonfire [ng/g].

It is obvious that PAHs may be formed in processed foods during heat treatment. Many studies have shown that high levels of PAHs result from high frying temperatures and are time-dependent [10]. The effect of grilling on the PAH8 concentration in white marshmallows is given in Figure 5. The concentrations of the compounds were higher when marshmallows were grilled in the oven (on average 175.8% ± 129.3% more, but the correlation between them was positive r^2^ > 0.8800, *p* = 0.0005). In this case, the temperature was higher and, more importantly, constant and not affected by gusts of wind.

**Figure 6:**
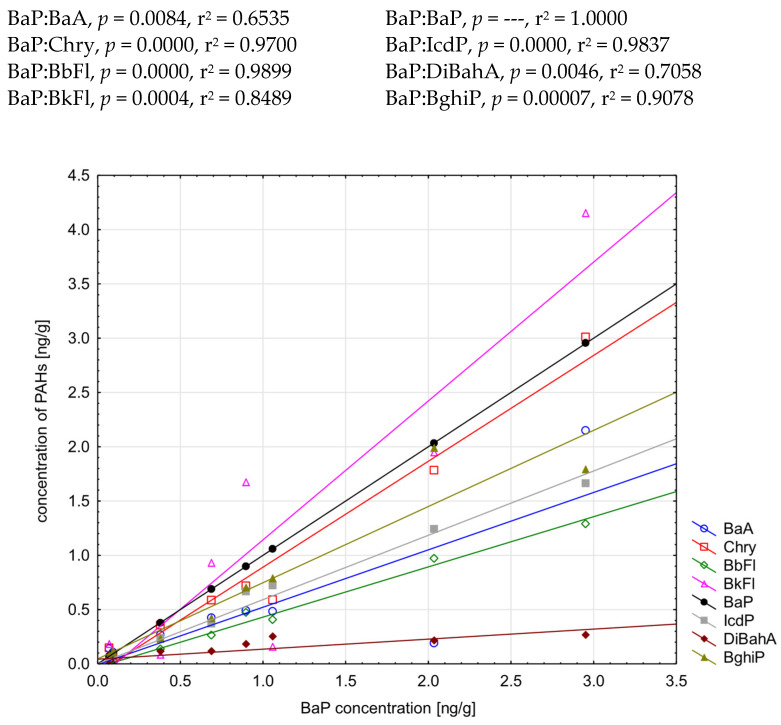
Correlations between BaP and PAH8 in grilled marshmallows.

Highly positive correlations between BaP and other carcinogenic PAH8 were observed in grilled marshmallows (r^2^ > 0.9000), except for BaA and DiBahA (Figure 6).

Research conducted on the use of BaP as a marker of exposure to PAHs in soil in the UK showed similar results [40]. Research results confirmed that benzo(a)pyrene alone induced only tumors of the alimentary tract, whereas PAH mixtures also caused other tumors [39].

Relatively high concentrations of anthracene, phenanthrene and pyrene were also found in the extracts analyzed using the GC-MS/MS technique. Compared to anthracene, we found a significantly higher concentration in the case of phenanthrene (6.74 ng/g in colored marshmallows; gGPY) and a ratio of 6.23 ± 1.87, although the correlation of these two PAHs was very high (r^2^ = 0.9808, *p* = 0.0001).

Pyrene is used to produce dyes, plastics and pesticides and to obtain other PAHs, such as benzo(a)pyrene. Exposure to pyrene may be caused by exposure to tobacco smoke as well as the consumption of food grown in soil contaminated by PAHs and the consumption of smoked fish, meat and grilled food. Its presence is also found in surface waters and drinking water [41]. Animal studies showed that exposure to pyrene developed nephropathy in mice. Additionally, changes in the blood and liver were reported [41].

The concentration of pyrene in grilled marshmallows was one or even two orders higher than that of other PAHs. The mean concentration of pyrene in white marshmallows grilled over the bonfire was 15.26 ± 5.36 ng/g. It was higher in marshmallows grilled in the oven (42.04 ng/g) and significantly higher (70.1 ± 37.13 ng/g) in colored marshmallows grilled over the bonfire.

**Figure 7:**
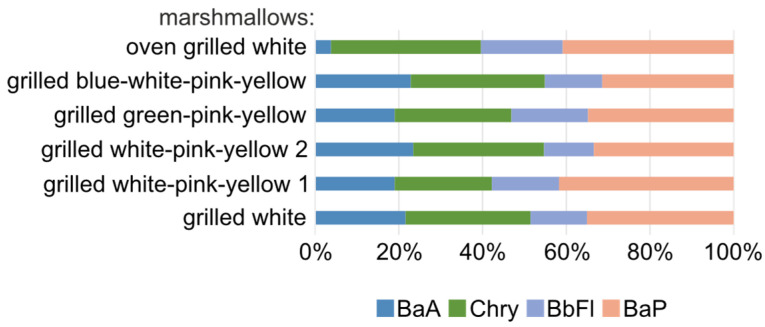
Percentage of PAHs in PAH4 in grilled marshmallows determined using the GC-MS/MS technique (recommended for determination in food by the EFSA).

The percentage of PAHs among the four compounds (Figure 7) most dangerous to human health in all grilled marshmallows was similar (more than in a study of Quing Liu et al. [32]). Among the PAH4 in marshmallows grilled over the bonfire, on average, the highest percentage was 30% for BaP and Chry. The highest average sum of PAH4 concentrations in grilled multi-colored marshmallows (gBWPY) was 9.40 ng/g, including 3.011 ng/g and 2.95 ng/g for BaP and Chry, respectively. Also, high values were obtained for marshmallows grilled in the oven, i.e., the sum of PAH4 was 4.98 ng/g, including 2.03 ng/g and 1.78 ng/g for BaP and Chry, respectively.

**Figure 8:**
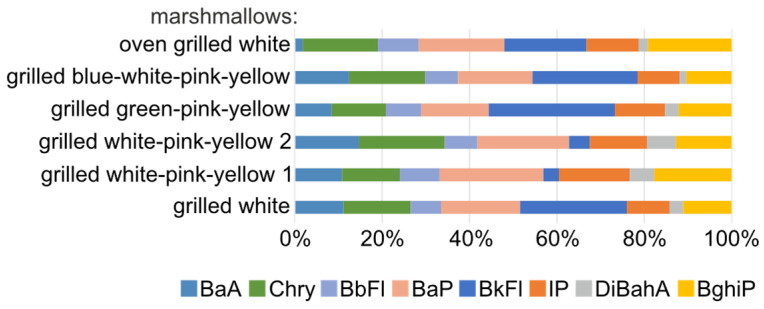
Percentage of PAHs in PAH8 in grilled marshmallows determined using the GC-MS/MS technique (recommended for determination in food by the EFSA).

Similarly, among the concentrations of PAH8 (Figure 8), the highest percentages were found for BaP, Chry and BghiP (19%, 16% and 14%, respectively), while the lowest percentage was found for DiBahA (4%).

In turn, among the PAH8, the average sum in grilled marshmallows was also the highest for multi-colored marshmallows grilled over the bonfire (17.27 ng/g) and then in grilled marshmallows in an oven (ogWHI; 10.37 ng/g), and the highest concentration (4.15 ng/g) was obtained for BkFl for multi-colored marshmallows grilled over a bonfire (gBWPY).

Considering the interdependence of the components of the PAH4 sum, the correlation coefficients were approximately >0.9900, and the lowest of which was 0.9843 for BbFl (see Table 4 below). In the PAH8 group, the correlations between PAHs in this study were positive and high (r^2^ > 0.9400). In turn, the value was the lowest for DiBahA (0.5061). It seems that in the assessment of exposure to PAHs in grilled marshmallows, only one of the compounds from the PAH2 group could be determined (r^2^ > 0.9900), e.g., Chry or BaP similar to PAHs in the soil during risk assessment in the UK [40].

Based on the available data on PAH content in various foods, the Food Authority estimated the health risks associated with exposure to PAHs in foods. Based on the average consumption of specific product groups, it was calculated that the most significant contribution to PAH exposure from the diet was the consumption of cereals and cereal products, vegetables, nuts, legumes, meat and meat products as well as fishery products. The estimated average exposure to PAHs in the EU countries for a person of 60 kg was 235 ng/day for benzo(a)pyrene and 1168 ng/day for PAH4. Special attention was paid to the high consumption of grilled foods, which leads to high exposure to PAHs [39].

Care for children’s health should include their exposure to PAHs from various sources. Therefore, it seems important to disseminate information that grilling produces compounds harmful to health. To the best of our knowledge, there are no data in the literature on the presence of PAHs in marshmallows. This product is occasionally but eagerly chosen by children and young adults. Although grilled marshmallows are not eaten on a daily basis, any additional exposure of children to mutagenic and carcinogenic compounds is unnecessary and should be avoided as much as possible.

## 3. Materials and Methods

### 3.1. Chemicals

#### 3.1.1. Reagents

Cyclohexane (ACS reagent ≥ 99%) and methanol (HPLC-grade) were obtained from Sigma Aldrich (Steinheim, Germany). Dichloromethane for liquid chromatography was obtained from Merck (Darmstadt, Germany), while toluene (analytical-reagent grade) was collected from Avantor™ Performance Materials (Gliwice, Poland). Water was obtained from a water purification system (Millipore, Vienna, Austria).

#### 3.1.2. PAHs

The polycyclic aromatic hydrocarbons (PAHs) mixture in benzene–methylene chloride (50:50) at a concentration of 2 mg/mL was obtained from Supelco (Bellefonte, PA, USA). The mixture contained 18 PAHs: acenaphthene, acenaphthylene, anthracene, benz(a)anthracene (BaA), benzo(b)fluoranthene (BbFl), benzo(k)fluoranthene (BkFl), benzo(ghi)perylene (BghiP), benzo(a)pyrene (BaP), chrysene (Chry), dibenz(a,h)anthracene (DiBahA), fluoranthene, fluorene, indeno(1,2,3-cd)pyrene (IP), 1-methylnaphthalene, 2-methylnaphthalene, naphthalene, phenanthrene and pyrene. The solution was diluted in toluene to prepare a standard stock solution (concentration of 40 µg/mL). Solutions at concentrations of 0.01, 0.05, 0.1, 0.5, 1, 5, and 10 µg/mL of toluene were prepared from the standard stock solution. These dilutions were used to define detection limits and make standard calibration plots.

#### 3.1.3. SPE Columns

Solid-phase extraction (SPE) columns (Bond Elut Si) containing 500 mg of silica gel (particle size: 40 µm) were obtained from Agilent (Santa Clara, CA, USA). The columns were conditioned by triple-washing with 3 mL of cyclohexane.

#### 3.1.4. HPTLC Plates

HPTLC Nano-SIL-PAH F_254_ (10 × 10 cm Macherey-Nagel (Düren, Germany) plates were used.

### 3.2. Marshmallows

Four packages (400 g each) of commercially available marshmallows were purchased from the local supermarket. The characteristics of marshmallows used in this study, as described on the packages, are included in Table 5.

#### Marshmallow Grilling

Marshmallows were grilled on an open fire using beech wood. No artificial kindling was used. All of them were grilled in the same manner, i.e., four pieces on a skewer grilled at the same height above the fire during the same period of time (on average about 12 g for 30 s) until their volume increased and the color was changed (ranging from caramel to brown). One portion of white marshmallows was grilled in an electric oven at 180 °C to obtain a similar effect to the flame of a bonfire. From 100 g of white marshmallows, an average of 96.3 g of grilled marshmallows was obtained, while from 100 g of colored marshmallows, an average of 95.71 g of grilled marshmallows was obtained.

### 3.3. Isolation of PAHs Fraction from Marshmallows

#### 3.3.1. Liquid–Liquid Extraction

Raw and grilled marshmallows were weighed, placed in a 500 mL wide-mouth bottle and diluted with deionized water. An amount of 300 mL of water was used for 100 g of marshmallows. The solution was stirred until the marshmallows were completely dissolved. Next, 80 mL of cyclohexane was added. After closing the bottle, the solution was subjected to ultrasound-assisted extraction in an ultrasonic washer (Advantage-Labor, Inc., Cypress, TX, USA) for 15 min at 37 °C. The solution was then transferred to a separatory funnel and after the separation of the layers, the cyclohexane fraction (upper layer) was transferred to a new beaker. The aqueous phase was subjected to ultrasound-assisted extraction twice more using 80 mL of cyclohexane each time. The cyclohexane fractions obtained as a result of three repeated liquid–liquid extraction processes were combined and evaporated using a vacuum evaporator to a volume of about 1 mL. The selection of the extraction procedure and the solvent was based on the analysis of PAHs in chocolate candies [42].

#### 3.3.2. Solid-Phase Extraction

Apart from PAHs, cyclohexane extracts from marshmallows may also contain other organic compounds, including dyes and oxygen or nitrogen derivatives of PAHs, usually formed during food thermal processing [43,44].

To avoid the influence of these components on the results of PAH determinations, cyclohexane extracts obtained from marshmallow samples were subjected to further purification using solid-phase extraction on columns filled with silica gel. This method was successfully used to separate PAH fractions from their polar derivatives when analyzing environmental samples with a much more complex matrix than marshmallow samples [45]. The extracts obtained as a result of liquid–liquid extraction were dissolved in 1 mL of cyclohexane applied to SPE-silica gel columns. The PAH fractions were eluted with 9 mL of cyclohexane, while the polar PAH derivatives were further eluted with dichloromethane (12 mL) and methanol (6 mL). The PAH derivatives were not the subject of this study. The cyclohexane extract obtained from the SPE step was dried under a gentle stream of gaseous nitrogen and re-dissolved in 250 µL of toluene before GC-MS analysis.

### 3.4. Preliminary Assessment of PAHs Using HPTLC Analysis

To compare the separated extracts from grilled and non-grilled marshmallows, the preliminary assessment was conducted using planar chromatography. For this purpose, planar separations were performed using HPTLC Nano-SIL-PAH F_254_ plates, in which the nano-silica layer had been impregnated with the electron acceptor caffeine [31]. PAH standards and cyclohexane extracts from grilled marshmallows were applied to chromatographic plates by Nanomat (Camag, Muttenz, Switzerland) equipped with 0.5 µL capillaries and then developed over a distance of 7 cm with dichloromethane in a horizontal chamber at −18 °C in the freezer. Visualization was performed under UV light at λ = 366 nm in a UV/Vis chamber (Camag).

### 3.5. Analysis of PAHs by Gas Chromatography–Mass Spectrometry (GC-MS/MS)

A gas chromatograph (Trace 1310 Thermo Scientific, Milano, Italy) with a triple quadrupole mass spectrometer (TSQ 9000, San Jose, CA, USA) was applied for qualitative–quantitative analysis. PAHs were separated on a TG-5MS GC capillary column (30 m × 0.25 mm; film thickness: 0.25 µm) from Thermo Scientific Waltham, MA, USA. The analysis of PAHs was performed as follows: splitless injection (2 min), helium flow rate of 1 mL/min; GC temperature program: 70 °C (2 min), 5 °C/min to 320 °C (5 min), temperature of the injector 270 °C, interface 320 °C, ion source 320 °C and electron ionization (EI) 70 eV. Cyclohexane extract solutions were injected into the column at 1 µL using a TriPlus RSH autosampler (Thermo Fisher Scientific, San Jose, CA, USA). Analyses were carried out in the full scan mode (mass range 50–550 Da) and in the selected reaction monitoring (SRM) mode, in which the precursor ions and product ions were selected as a result of an automatically performed experiment for a PAH standard solution. The selected PAH ions with collision energy in qualitative and quantitative analysis by GC-MS/MS in the SRM are given in Table 6.

The qualitative analysis of PAHs involved the comparison of mass spectra and retention times of the compounds identified in marshmallow sample extracts and standards. Identification was performed using Mainlib and NIST (National Institute of Standards and Technology in the USA) mass spectral databases and Chromeleon software (version 7.2.10, Thermo Fisher, USA). The obtained mass spectra of PAHs were dominated by their molecular ions M^+^.

## 4. Conclusions

A multi-step analytical scheme was used to determine PAHs in grilled marshmallows by GC-MS/MS technique with sufficient efficiency and satisfactory repeatability. No presence of PAHs was found in raw marshmallows. Higher concentrations of PAHs were determined in multi-colored than in white marshmallows. The results suggest a relatively high exposure to carcinogenic PAHs in the population of young people and children consuming grilled marshmallows on a regular basis. The profiles of PAH concentrations in grilled marshmallows were similar and did not depend on the method of grilling.

The human body has mechanisms for detoxifying PAHs. It is essential to reduce the exposure to PAHs among young people. The lack of public awareness related to the exposure to harmful compounds, including carcinogenic substances, which are formed in thermally processed food, is alarming.

## Figures and Tables

**Table 1:** Limits of detection (LOD) and quantitation (LOQ) of PAH in standard solution.

Compound	LOD ng/mL	LOQ ng/mL
Phenanthrene	15.0	50.0
Anthracene	3.0	10.0
Pyrene	15.0	50.0
BaA	15.0	50.0
Chry	15.0	50.0
BbFl	15.0	50.0
BkFl	15.0	50.0
BaP	15.0	50.0
IP	25.0	80.0
DiBahA	25.0	80.0
BghiP	25.0	80.0

**Table 2:** Concentrations of PAHs in grilled and non-grilled marshmallows * [ng/g] (gWHI—grilled white; gWPY 1—grilled white–pink–yellow; gWPY 2—grilled white–pink–yellow; gGPY—grilled green–pink–yellow; BWPY—grilled blue–white–pink–yellow; ogWHI—oven grilled white; WPY 2—raw white–pink–yellow; WPY 1—raw white–pink–yellow; GPY—raw green–pink–yellow).

Sample	BaA	Chry	BbFl	BkFl	BaP	IcdP	DiBahA	BghiP
gWHI	0.424	0.586	0.265	0.932	0.688	0.37	0.119	0.423
gWPY 1	0.482	0.589	0.407	0.159	1.059	0.721	0.252	0.789
gWPY 2	0.266	0.354	0.135	0.086	0.379	0.237	0.119	0.230
gGPY	0.490	0.718	0.471	1.675	0.897	0.666	0.182	0.702
BWPY	2.149	3.011	1.289	4.152	2.953	1.661	0.266	1.792
ogWHI	0.189	1.783	0.972	1.947	2.034	1.240	0.217	1.989
WPY 2 *	0.067	0.069	0	0.093	0.094	0	0	0.102
WPY 1 *	0.107	0.052	0	0.121	0.095	0	0	0.118
GPY *	0.140	0.145	0	0.183	0.071	0	0	0.074

**Table 3:** The BaP to PAH8 ratio in grilled marshmallows.

Ratio of PAHs	Mean	±SD
BaP:BaA	1.26	0.32
BaP:Chry	1.69	0.34
BaP:BbFl	2.44	0.35
BaP:BkFl	1.79	2.02
BaP:IP	1.61	0.21
BaP:DiBahA	5.84	3.09
BaP:BghiP	1.51	0.18

**Table 4:** Correlations between PAH2, PAH4 and PAH8 sums and individual PAHs.

Sum of PAH8 vs	r^2^	Sum of PAH4 vs	r^2^	Sum of PAH2 vs	r^2^
BaA	0.9823	BaA	0.9960	BaP	0.9918
BaP	0.9812	BaP	0.9911	Chry	0.9927
BbFl	0.9933	BbFl	0.9843		
Chry	0.9853	Chry	0.9938		
BkFl	0.9407				
IcdP	0.9596				
DiBahA	0.5061				
BghiP	0.9575				

**Table 5:** The characteristics of marshmallows.

Sample	Name of the Product	Color	Ready to Grill Yes/No	Composition per 100 g
1A, 1B	K-Classic Marshmallows	pink, yellow	no	carbohydrates (80 g), including sugars (68 g); protein (4 g)
B1, B2 and 4	MCENNEDY, American Way BBQ Marshmallows	white	yes	carbohydrates (80.3 g), including sugars (60.4 g); protein (3.4 g); fat (0.1 g)
2 and 5	Twisted Jojo marshmallow with a fruit vanilla flavor	white, yellow, pink	no	carbohydrates (76.42 g), including sugars (73.96 g); protein (4.28 g); fat (0.21 g); salt (0.15 g); fiber (0.10 g)
3	Haribo Chamallows Mallow Mania	pink, yellow, green	no	carbohydrates (80 g), including sugars (68 g); protein (3.5 g)
6	Haribo Chamallows the Smurfs Family	white, yellow, pink and blue	no	carbohydrates (80 g), including sugars (68 g); protein (3.5 g)

**Table 6:** The parameters of PAH analysis by GC-MS/MS in the SRM mode.

Compound	Retention Time [min]	Quantitative Ion Pair		Qualitative Ion Pair	
		Precursor Ion (*m*/*z*) → Product Ion	Collision Energy (eV)	Precursor Ion (*m*/*z*) → Product Ion	Collision Energy (eV)
Phenanthrene	25.50	178.1→128.1	30	178.1→152.1	20
Anthracene	25.71	178.1→150.1	45	178.1→152.1	20
Pyrene	32.01	202.1→200.1	35	202.1→201.1	20
BaA	37.69	228.1→225.2	45	228.1→226.1	30
Chry	37.86	228.1→226.1	30	228.1→202.1	25
BbFl	42.41	252.1→250.1	30	252.1→226.1	25
BkFl	42.51	252.1→250.1	35	252.1→226.1	25
BaP	43.64	252.1→250.1	35	252.1→226.1	25
IP	47.74	276.1→274.1	35	276.1→275.2	25
DiBahA	47.89	278.1→276.1	30	278.1→252.1	25
BghiP	48.55	276.1→274.1	40	276.1→275.2	25

## Data Availability

The raw data supporting the conclusions of this article will be made available by the authors on request.
